# Population Structure, Genetic Diversity and Differentiation of *Triplophysa tenuis* in Xinjiang Tarim River

**DOI:** 10.3389/fgene.2022.860678

**Published:** 2022-03-03

**Authors:** Bin Huo, Xuan Liu, Shengao Chen, Jieya Liu, Qiong Zhou, Jianzhong Shen, Dapeng Li, Rong Tang, Jing Chen, Xiaoyun Zhou

**Affiliations:** ^1^ College of Fisheries, Huazhong Agricultural University, Wuhan, China; ^2^ College of Animal Science, Tarim University, Alar, China

**Keywords:** Triplophysa tenuis, tarim river, population structure, genetic diversity, genetic differentiation

## Abstract

*Triplophysa tenuis* is an important indigenous fish in the Xinjiang Tarim River. In this study, we collected 120 *T. tenuis* individuals from 8 *T. tenuis* populations in the Tarim River. Through genotyping-by-sequencing (GBS), a total of 582,678,756 clean reads were generated for all the genotypes, and after quality filtering, 595,379 SNPs were obtained for the population genetic analyses. Multiple genetic parameters showed that the 8 *T. tenuis* populations had high genetic diversity. Phylogenetic tree analysis indicated that all *T. tenuis* individuals were divided into five branches, the individuals from the north of Tarim River were grouped into cluster 1 (SF and WS) and cluster 3 (DWQ, TKX, and KZE), while the AETS, WLWT and LF individuals from the south of Tarim River were clustered into cluster 2. The result was consistent with the admixture analysis, which supported that the 8 *T. tenuis* populations were clustered into three subgroups. Furthermore, the pairwise *F*_*ST*_ values and genetic distance indicated that there was a large genetic differentiation between WS and other *T. tenuis* populations. Collectively, this study provides valuable genome-wide data for the conservation of natural *T. tenuis* populations in the Tarim River.

## Introduction

*Triplophysa tenuis*, which belongs to *Triplophysa*, Nemacheilidae, and Cypriniformes, inhabits the backwater of rivers over sandy or muddy bottoms. It is recognized as an important indigenous fish with certain economic value in southern Xinjiang Tarim River, such as Kaidu River, Aksu River (Xie et al., 2015). The feeding habit of *T. tenuis* is omnivorous, partial to carnivorous, mainly feeding on benthos, fish, shrimp and insect larvae, followed by algae and organic debris (Tursun et al., 2005). The genus *Triplophysa* (Plateau loach) is a special group in the Qinghai-Tibet Plateau, which has strong adaptability to the highland environment. In recent years, affected by natural and anthropic factors, the Tarim River has experienced runoff curtailment and river desiccation, accompanied by river salinization, biodiversity decrease, and ecosystem service loss (Chen et al., 2011). As a result, the population size of *T. tenuis* has been diminishing and it is critically endangered due to its habitat limited to the upstream of the Tarim River. Although previous studies have reported the reproductive, morphological, and other biological characteristics of *T. tenuis*, the genetic diversity and genetic differentiation of *T. tenuis* populations are still unclear (Tursun et al., 2005; Liu et al., 2021). Understanding the genetic diversity and genetic differentiation among *T. tenuis* populations is essential for designing conservation and management strategies.

Traditional molecular markers play a crucial role in population genetics analysis, improving the understanding of complex quantitative traits, and facilitating marker-assisted breeding (Zhong et al., 2019). In the past decades, the most commonly used molecular markers include random amplified polymorphic DNA (RAPD), mitochondrial DNA sequences, simple sequence repeat (SSR), and single nucleotide polymorphism (SNP) (Younis et al., 2020). Notably, SNPs have become the preferred markers for genetic studies due to their unique characteristics, such as unbiased distribution, biallelic properties, and availability in the whole genome (Islam et al., 2021). Next-generation sequencing (NGS) technologies have been recently conducted for molecular marker development, population genetic analysis, and molecular breeding. For the species without a reference genome, reduced-representation genome sequencing (RRGS) can be utilized to obtain genome-wide genetic variation information. According to the different library construction strategies, RRGS is divided into reduced-representation libraries (RRL), restriction-site associated DNA (RAD), and genotyping-by-sequencing (GBS). Among them, GBS method reduces genome complexity and allows the discovery of genome-wide SNPs with a lower error rate based on restriction enzymes (REs) (Wang et al., 2020). GBS is the most widely used in aquatic species based on NGS with high-throughput genotyping and low per-sample, such as *Oryzias latipes* (Katsumura et al., 2019), *Cynoglossus semilaevis* (Zhang et al., 2020), and *Misgurnus anguillicaudatus* (Yi et al., 2019).

Large-scale genotyping at the whole genome level is becoming more and more important for population genetic studies (Zhang et al., 2019). Herein, we collected 8 *T. tenuis* populations and designed this study by the GBS method with the following objectives: 1) investigate fine-scale genetic variations of *T. tenuis* populations by the GBS database; 2) provide genomic evidence on the population structure of *T. tenuis* by a phylogenetic tree, admixture analysis, and principal component analysis; 3) determine the genetic diversity of 8 *T. tenuis* populations by detecting the genetic parameters, as well as explore the population genetic differentiation of 8 *T. tenuis* populations by the pairwise *F*_*ST*_ and genetic distance. This study is essential to the germplasm resource conservation of *T. tenuis* in Xinjiang Tarim River, as well as provides a valuable reference for genome-assisted breeding of *T. tenuis*.

## Materials and Methods

### Samples Information

**FIGURE 1:**
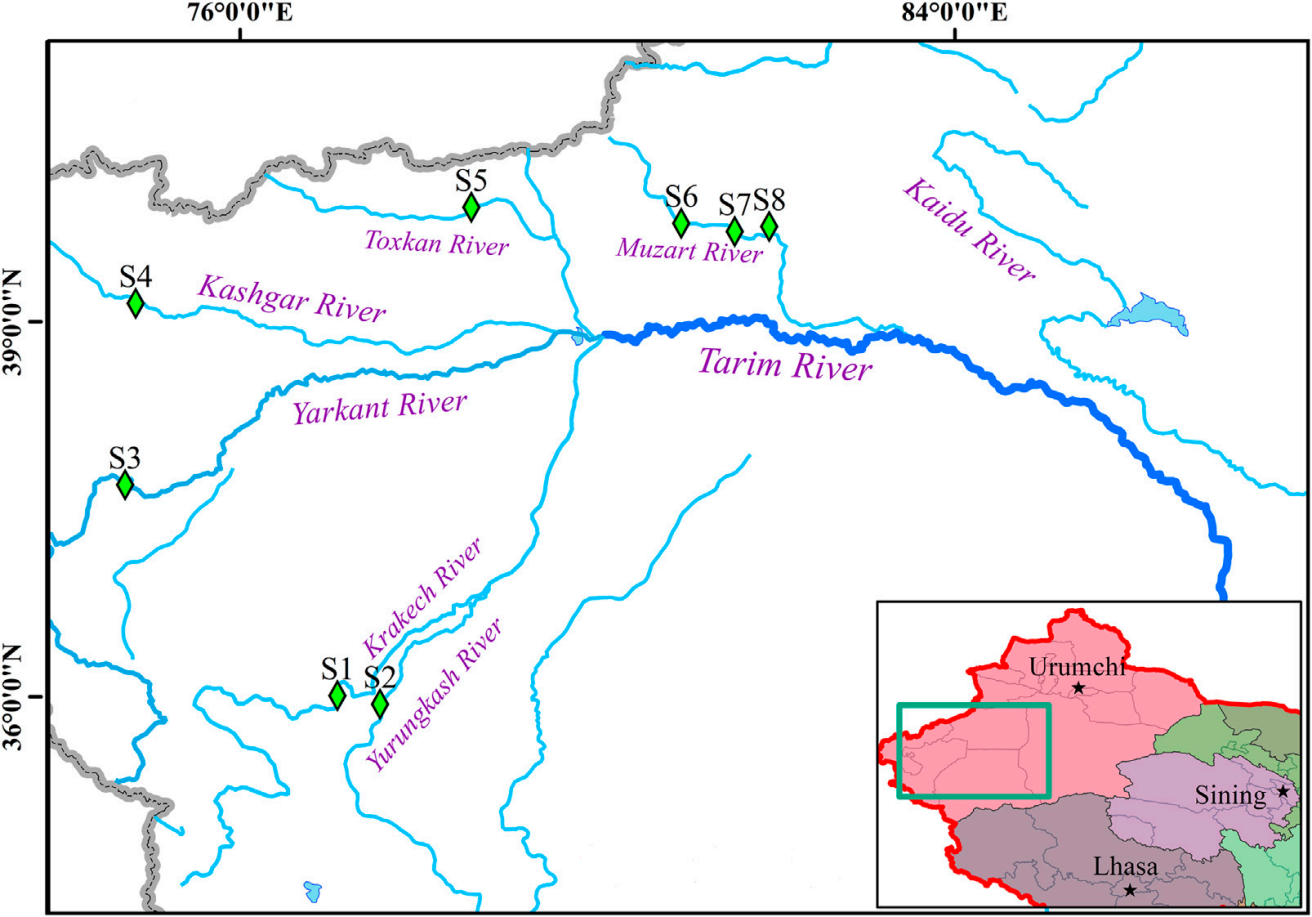
The distribution of *T. tenuis* populations in the branches of the Tarim River. The blue lines represent the rivers. The green square symbols indicate the sampling locations. S1, WLWF; S2, LF; S3, AETS; S4, SF; S5, WS; S6, DWQ; S7, TKX; S8, KZE.

In this study, a total of 120 *T. tenuis* samples were collected from the branches of the Tarim River, including the Krakech River (WLWT, n = 15), the Yurungkash River (LF, n = 15), the Yarkant River (AETS, n = 15), the Kashgar River (SF, n = 15), the Toxkan River (WS, n = 15), the Muzart River (DWQ, n = 15; TKX, n = 15; KZE, n = 15) in 2021 (Figure 1). The fin tissues of all samples were collected and preserved in 95% ethanol. Total DNA was extracted using a DNA extraction kit (Tiangen, Beijing). The quality and quantity of DNA were detected by NanoDrop 2000 spectrophotometer (Thermo Scientific, Wilmington, DE, United States), agarose gel electrophoresis and Agilent 2,100 Bioanalyzer (Agilent, Santa Clara, CA, United States).

### Library Construction and Quality Control

The library was constructed by Super-GBS method (Qi et al., 2018) as follows: DNA was digested by PstI HF/MspI; T4 ligase was used to add linker and barcode at both ends of the digested fragment; 300–700 BP fragments were recovered by adjusting the volume ratio of magnetic bead solution to the connecting product; The recovered fragments were amplified by PCR with high fidelity enzyme. The mixed library was sequenced by Illumina NovaSeq platform with 150 paired-end. We used stacks (Catchen et al., 2013) software and split the offline data according to barcode and enzyme digestion site information to obtain raw reads of each sample. Moreover, the raw reads were filtered with the fastp program (Chen et al., 2018), and the quality filtering standards were as follows: remove the joint sequence; remove reads with N (non AGCT) base greater than or equal to 5; remove reads with an average base mass value less than 20.

### Genome Construction and Variation Detection

The ustacks program in Stacks software (Catchen et al., 2013) was used to cluster the sequenced reads of each sample, while cstacks, sstacks, tsv2bam, and gstacks programs were used for genome construction and variation detection. In addition, we used vcftools software (Danecek et al., 2011) to filter the SNP typing results. The filtering conditions were as follows: non-second alleles were excluded; The number of reads support (DP) was not less than 4; the loci with MAF less than 0.01 were excluded; the loci with SNP typing deletion rate higher than 20% were excluded.

### Population Genetic Structure Analysis

To explore the population structure of *T. tenuis* with different geographical distribution, admixture software (v1.3.0) (Alexander et al., 2009) was used to cluster all samples with a bayesian approach. The *K* value was determined according to ten-fold cross-validation. Pong software (Behr et al., 2016) was used to cluster the repeated results of each *K* value. The phylogenetic tree was constructed by the neighbor-joining (NJ) method, the distance matrix was calculated by treebest software (Ruan et al., 2008). The gcta software (Yang et al., 2011) was used to perform principal component analysis (PCA) by genome-wide SNPs information, and the scatter plotting was performed with the first three principal components using the “ggplot2” package in R (Ginestet, 2011).

### Genetic Differentiation and Genetic Diversity Analyses

The genetic differentiation coefficient (*F*_*ST*_) between populations was calculated using the R package genepop (Rousset, 2008). The *F*_*ST*_ values ranging from 0 to 0.05, 0.05 to 0.15, and 0.15 to 0.25 indicate that there are no genetic, moderate, and large differentiations among populations, respectively (Wright, 1965). The genetic distance (DR) between populations was estimated by *F*_*ST*_ value (Reynolds et al., 1983). Furthermore, several genetic indicators were used to investigate the genetic diversity of 8 *T. tenuis* populations, including Hardy-Weinberg equilibrium *p*-value (*HW-P*), Hardy-Weinberg (*He*), observed heterozygosity (*Ho*), polymorphism information content (*PIC*), effective number of alleles (*Ne*), observed number of alleles (*Na*), nucleotide diversity (*Pi*). The vcftools software was used to calculate these genetic indicators (Danecek et al., 2011).

## Results

### Identification and Screening of SNP Markers

In this study, a total of 120 *T. tenuis* samples were sequenced and genotyped using GBS method. The constructed reference genome contains 572,808 sequences. After filtering out the raw reads, the total clean reads for all the genotypes were 582,678,756 with the average reads per sample being 4,855,656 (Supplementary Table S1). The average clean reads percent for all samples was 96.88%, and the lowest and highest number of reads was 3,240,046 and 7,040,766, respectively. The content of clean bases ranged from 0.47 G to 1.03 G, the average GC content was 44.08%, and the percent of clean base varied from 92.82 to 97.82%. After processing the raw reads via the GBS pipeline, we obtained a total of 595,379 SNPs using the VCF filtering control thresholds for further genetic analysis. The average sequencing depth of all samples was 16.28 X. Clean reads of each sample demonstrated high Q20 (>95.68%) and Q30 (>89.24%), indicating the high quality of sequencing data.

### Population Genetic Structure Analysis of *T. tenuis* Populations

**FIGURE 2:**
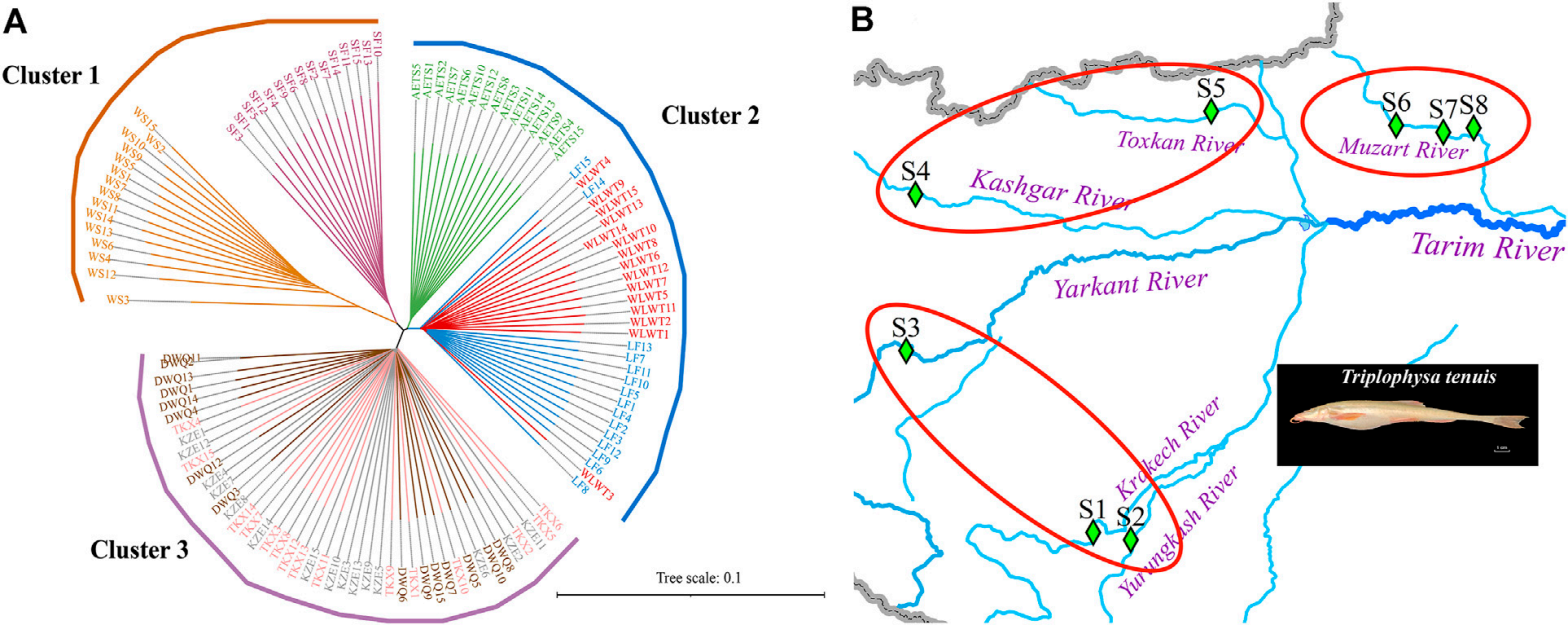
Phylogenetic tree analysis of 8 *T. tenuis* populations. **(A)** Neighbor-Joining tree based on SNP data of 120 *T. tenuis* individuals. **(B)** Phylogenetic tree results corresponding to geographical location. The blue lines represent the rivers. The green square symbols indicate the sampling locations. S1, WLWF; S2, LF; S3, AETS; S4, SF; S5, WS; S6, DWQ; S7, TKX; S8, KZE.

**FIGURE 3:**
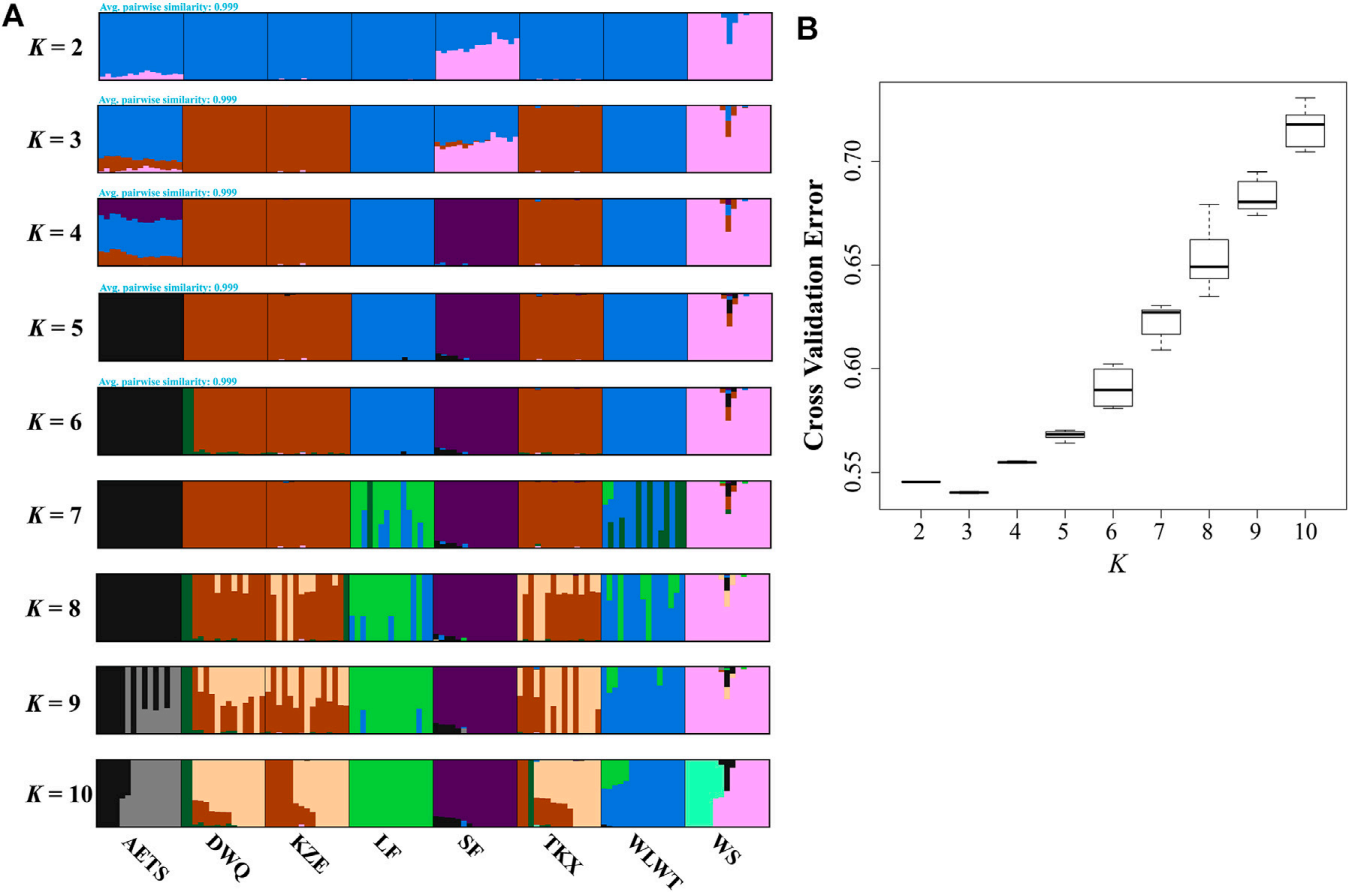
Population genetic structure based on SNP data of 120 *T. tenuis* individuals. **(A)** Clustering information for different populations when *K* = 2–10; **(B)** The CV error varies among *K* values.

To investigate the grouping of 8 *T. tenuis* populations, we constructed the NJ tree of the 120 individuals, which indicated that different geographical populations of *T. tenuis* could be divided into five branches (Figure 2A). The individuals from WS and SF were clustered into cluster 1. The individuals from WLWT and LF were grouped into one subgroup and clustered into cluster 2 with the AETS individuals. Additionally, the KZE, DWQ, and TKX individuals were tightly clustered together to form cluster 3 (Figure 2B). The scatter plots of PCA showed that the WS individuals were separate from the SF individuals and that the DWQ, KZE and TKX individuals are close to each other, but distinct from AETS, WLWT and LF individuals (Supplementary Figure S1). Pong analysis was used to perform the genetic clusters for all individuals (*K* = 2–10), and nine independent runs for each *K* value were conducted (Figure 3A). Admixture analysis indicated that the minimizing *K* value was 3 (Figure 3B), suggesting that all *T. tenuis* individuals could be divided into three subgroups, which was consistent with the result of the phylogenetic tree. Therefore, it was revealed that the individuals from SF and WS showed different ancestry information compared with the individuals from the DWQ, KZE and TKX, and the individuals from the AETS, LF and WLWT.

### Genetic Differentiation Among *T. tenuis* Populations

**FIGURE 4:**
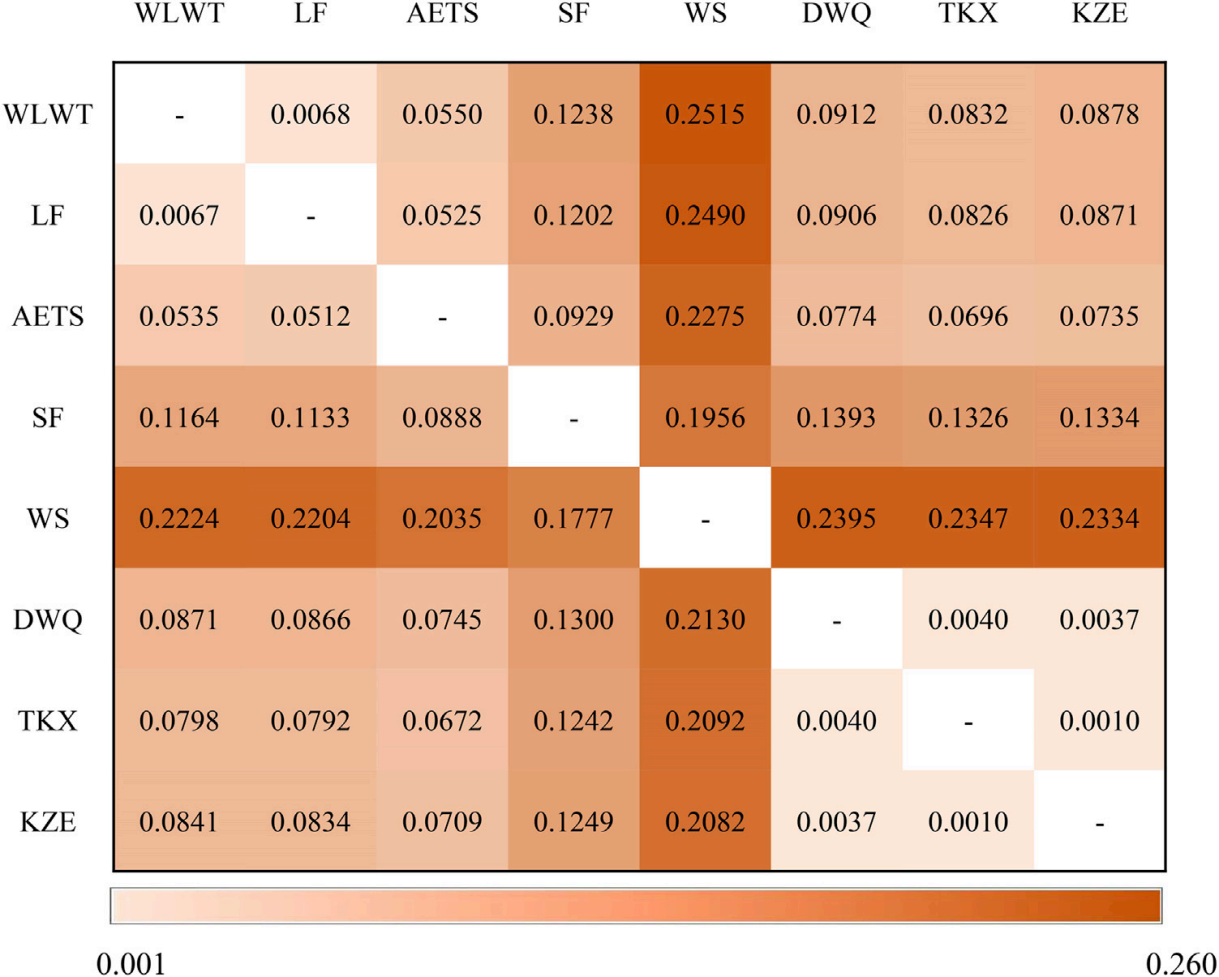
Genetic differentiation *F*_*ST*_ value (below diagonal) and genetic distance (above diagonal).

The genetic differentiation among the *T. tenuis* populations was identified by pairwise *F*_*ST*_ values and genetic distance (Figure 4). The *F*_*ST*_ values between pairs of the 8 *T. tenuis* populations varied from 0.0010 (KZE with TKX) to 0.2224 (WLWT with WS), with an overall *F*_*ST*_ value (0.1126), suggesting a moderate genetic differentiation among these *T. tenuis* populations. Notably, we found that the *F*_*ST*_ values ranged from 0.1777 (WS with SF) to 0.2224 (WS with WLWT), indicating that there was a large genetic differentiation between WS population and the other *T. tenuis* populations. Moreover, the genetic distance between pairs of the 8 *T. tenuis* populations ranged from 0.0010 (KZE with TKX) to 0.2515 (WLWT with WS). Likewise, there was a large genetic distance value between WS population and the other 7 *T. tenuis* populations, indicating an obvious genetic differentiation. By these analyses, we observed that there was a large genetic differentiation between WS and WLWT, a low genetic differentiation between KZE and TKX.

### Genetic Diversity of *T. tenuis* Populations

The genetic parameters are shown in Supplementary Table S2. The *HW-P* index showed the relationship between allele frequency and genotype frequency. We found that *HW-P* values ranged from 0.8495 (WS) to 0.8777 (WLWT), indicating that all populations reached genetic equilibrium. The *He* index represents the expected value of heterozygosity based on Hardy Weinberg equilibrium, which depends only on allele frequency. The result showed that *He* values ranged from 0.1298 (WLWT) to 0.1450 (WS), with an average of 0.1359. The *Ho* index indicates the proportion of heterozygotes observed in a population. We observed that *Ho* values ranged from 0.1198 (WLWT) to 0.1307 (SF), with an average of 0.1245. The *PIC* is an indicator of polymorphism level. The result showed that *PIC* values ranged from 0.1085 (WLWT) to 0.1214 (WS), with an average of 0.1139. Additionally, *Ne*, *Na* and *Pi* indices are the basic parameters of genetic diversity, the result indicated that the *Ne* values ranged from 1.2011 (WLWT) to 1.2268 (SF) with an average of 1.2100, and the *Na* values ranged from 1.5584 (WLWT) to 1.6172 (WS) with an average of 1.5931. Moreover, the *Pi* values ranged from 0.1348 (WLWT) to 0.1508 (WS), with an average of 0.1411.

## Discussion

The harsh environment, characterized by high altitude, cold habitat and poor nutrition in the Qinghai-Tibet Plateau, has brought severe challenges to local species (Yang et al., 2015). Nevertheless, as the representative indigenous species in the Qinghai-Tibet Plateau, *Triplophysa* fishes can well adapt to these severe natural conditions (Chen et al., 2020). Given their taxonomic status, evolutionary process, geographical distribution and biological characteristics, *Triplophysa* genus offers an attractive study model for the fish phylogeny, geological change, life evolution, and extreme environmental adaptation (Chen et al., 2020; Wu et al., 2020). In recent years, due to water diversion, irrigation and climatic change, coupling factors, including the lower reach dry-out, river salinization, habitat fragmentation, etc., drove *T. tenuis* to only inhabit the upper reaches of this river, and caused its population decline (Yaning et al., 2009). To investigate the genetic diversity and genetic structure of *T. tenuis* populations, we collected the *T. tenuis* individuals from five sampling points (SF, WS, DWQ, TKX and KZE) in the north of Tarim River, and three sampling points (WLWT, LF, and AETS) in the south of Tarim River. Through population genetic structure analysis, we found that the individuals from the south of Tarim River were clustered together, the individuals from the north of Tarim River were divided into two subgroups according to the geographical isolation.

Previous studies have revealed that geographical distance, ecological or environmental differences can bring about genetic isolation of populations and reduce the rate of successful migration (Chen and Wang, 2021; Gai et al., 2021). We found that the WS (Toxkan River) and SF individuals (Kashgar River) differentiated obviously and formed two separate clusters in cluster 1, and the AETS individuals (Yarkant River) differentiated with WLWT (Krakech River) and LF individuals (Yurungkash River) in cluster 2. The sampling points of Krakech River and Yurungkash River are relatively close in location, the individuals from WLWT showed a closer affinity to the LF. Additionally, the overall *F*_*ST*_ values indicated that there was a moderate gene differentiation among 8 *T. tenuis* populations, indicating that the genetic exchange among these *T. tenuis* populations was very restricted. Likewise, very large pairwise *F*_*ST*_ values were also estimated among *T. yarkandensis* populations in the Tarim River (Zhou et al., 2021). Tarim River is a typical seasonal and high salinity river, with an extremely fragile ecosystem and underdeveloped aquaculture. Thus, it is limited to mediated the genetic exchange among most populations mediated by agriculture activity. In the past decades, due to the influence of natural and anthropic factors, many branch rivers have successively lost surface water contact with the mainstream of the Tarim River. Therefore, it is inferred that the fragmentation of the Tarim River and its branch basins may be one of the important reasons for hindering the gene exchange among different *T. tenuis* populations.

Tarim River is the largest inland river in China, formed by the confluence of 114 rivers of 9 major water systems (Chen et al., 2007). The Krakech River is one of the main tributaries of the Hotan River, and has serious evaporation and seepage due to the middle reaches of this river crossing the Taklimakan Desert. Tashkurgan River is one of the main tributaries of the Yarkand River, but most of the water in the Yarkand River has been introduced into the reservoir since the Xiaohaizi Reservoir was constructed. Hence the two rivers only inject water into the mainstream of the Tarim River during the flood period. At present, Aksu River is the main water source of the Tarim River and supplies water to the Tarim River all year round (Hartmann et al., 2016). Herein, we collected the WS individuals from Toxkan River, which originates from the South Tianshan Mountains on the border between China and Kyrgyzstan, and merges with the Kumarak River to form the Aksu River. According to the pairwise *F*_*ST*_ values and genetic distance, we found that WS population had a large genetic differentiation with other *T. tenuis* populations. Moreover, it was indicated that WS population presented a higher genetic diversity. Therefore, it is suggested that the abundant water and the suitable habitat environment of the Tarim River have contributed to the formation of the unique genetic characteristics of *T. tenuis* (i.e., high genetic variation). Importantly, coupled with the fragmentation of the Tarim River, it may increase the possibility of outbreeding decline.

In conclusion, our study represented an important step in better understanding the genetic diversity and differentiation of *T. tenuis* within the Tarim River ecosystem, which is essential to develop the population management unit. Further, we envision that artificial propagation and releasing are conducted to restore the *T. tenuis* population in the Tarim River ecosystem. The genome-wide molecular markers can be available to establish a parent genetic information database and select an appropriate population for proliferation and release, which can not only be conducive to the restoration, but also avoid the genetic bottleneck effect and prevent genetic drift. Meanwhile, to maintain genetic diversity and avoid depletion of genetic resources, the genetic consequences of releasing the *T. tenuis* populations into the wild need to be evaluated.

## Data Availability

The datasets presented in this study can be found in online repositories. The names of the repository/repositories and accession number(s) can be found below: https://figshare.com/articles/dataset/Population_genetic_analysis_of_Triplophysa_tenuis/19126847/1.
