# Supplementary material for: Population Structure, Genetic Diversity and Differentiation of Triplophysa tenuis in Xinjiang Tarim River
Source: Front Genet. 2022 Mar 3;13:860678. doi: 10.3389/fgene.2022.860678 (PMC8927061; doi:10.3389/fgene.2022.860678)
Supplement: Supplementary file 1 [file DataSheet1.ZIP › Supplementary materials/Figure S1.docx]

**Figure S1. Principal component analysis (PCA) of eight *T. tenuis* populations.** (A) Scatter plot of PC1 and PC2; (B) Scatter plot of PC1 and PC3.
