# Supplementary material for: Population Structure, Genetic Diversity and Differentiation of Triplophysa tenuis in Xinjiang Tarim River
Source: Front Genet. 2022 Mar 3;13:860678. doi: 10.3389/fgene.2022.860678 (PMC8927061; doi:10.3389/fgene.2022.860678)
Supplement: Supplementary file 1 [file DataSheet1.ZIP › Supplementary materials/Table S1.docx]

Table S1. Quality control summary

| Samples | Raw Reads | Clean Reads | Clean Reads Percent | Raw_Base | Clean_Base | Clean Base Percent | GC Content | >Q20 | >Q30 |
| --- | --- | --- | --- | --- | --- | --- | --- | --- | --- |
| AETS1 | 4758092 | 4645156 | 97.63% | 699439524(0.70G) | 678192776(0.68G) | 96.96% | 44.05% | 96.96% | 91.46% |
| AETS10 | 4035854 | 3907732 | 96.83% | 593270538(0.59G) | 570528872(0.57G) | 96.17% | 44.11% | 96.63% | 90.72% |
| AETS11 | 5247228 | 5163978 | 98.41% | 771342516(0.77G) | 753940788(0.75G) | 97.74% | 43.81% | 96.86% | 91.28% |
| AETS12 | 5619064 | 5527252 | 98.37% | 826002408(0.83G) | 806978792(0.81G) | 97.70% | 43.81% | 96.86% | 91.29% |
| AETS13 | 4841146 | 4721300 | 97.52% | 711648462(0.71G) | 689309800(0.69G) | 96.86% | 44.12% | 96.91% | 91.37% |
| AETS14 | 5210378 | 5092650 | 97.74% | 765925566(0.77G) | 743526900(0.74G) | 97.08% | 44.36% | 96.97% | 91.48% |
| AETS15 | 4716524 | 4621378 | 97.98% | 693329028(0.69G) | 674721188(0.67G) | 97.32% | 43.66% | 96.76% | 91.06% |
| AETS2 | 5036896 | 4907802 | 97.44% | 740423712(0.74G) | 716539092(0.72G) | 96.77% | 44.43% | 96.96% | 91.47% |
| AETS3 | 4890646 | 4786724 | 97.88% | 718924962(0.72G) | 698861704(0.70G) | 97.21% | 43.94% | 96.93% | 91.44% |
| AETS4 | 5228274 | 5092298 | 97.40% | 768556278(0.77G) | 743475508(0.74G) | 96.74% | 44.17% | 97.05% | 91.70% |
| AETS5 | 5104620 | 4974252 | 97.45% | 750379140(0.75G) | 726240792(0.73G) | 96.78% | 44.15% | 96.99% | 91.53% |
| AETS6 | 5070932 | 4975752 | 98.12% | 745427004(0.75G) | 726459792(0.73G) | 97.46% | 44.14% | 96.80% | 91.13% |
| AETS7 | 5075222 | 4985630 | 98.23% | 746057634(0.75G) | 727901980(0.73G) | 97.57% | 44.11% | 96.79% | 91.07% |
| AETS8 | 4928658 | 4816130 | 97.72% | 724512726(0.72G) | 703154980(0.70G) | 97.05% | 43.85% | 96.76% | 91.07% |
| AETS9 | 5077404 | 4964748 | 97.78% | 746378388(0.75G) | 724853208(0.72G) | 97.12% | 44.31% | 96.81% | 91.14% |
| DWQ1 | 5216966 | 5056680 | 96.93% | 766894002(0.77G) | 738275280(0.74G) | 96.27% | 44.05% | 96.75% | 91.30% |
| DWQ10 | 5578284 | 5453952 | 97.77% | 817218606(0.82G) | 796276992(0.80G) | 97.44% | 43.81% | 96.43% | 90.62% |
| DWQ11 | 5889026 | 5736916 | 97.42% | 862742309(0.86G) | 837589736(0.84G) | 97.08% | 43.99% | 96.69% | 91.16% |
| DWQ12 | 5352264 | 5202300 | 97.20% | 784106676(0.78G) | 759535800(0.76G) | 96.87% | 43.64% | 96.65% | 91.08% |
| DWQ13 | 6163358 | 6009616 | 97.51% | 902931947(0.90G) | 877403936(0.88G) | 97.17% | 43.94% | 96.58% | 90.91% |
| DWQ14 | 5726284 | 5571926 | 97.30% | 838900606(0.84G) | 813501196(0.81G) | 96.97% | 44.07% | 96.94% | 91.66% |
| DWQ15 | 6194086 | 6059718 | 97.83% | 907433599(0.91G) | 884718828(0.88G) | 97.50% | 43.95% | 96.82% | 91.41% |
| DWQ2 | 5343092 | 5202470 | 97.37% | 785434524(0.79G) | 759560620(0.76G) | 96.71% | 43.88% | 96.74% | 91.27% |
| DWQ3 | 5194968 | 5026528 | 96.76% | 763660296(0.76G) | 733873088(0.73G) | 96.10% | 44.18% | 96.89% | 91.56% |
| DWQ4 | 6800782 | 6616670 | 97.29% | 996314563(1.00G) | 966033820(0.97G) | 96.96% | 43.71% | 96.46% | 90.71% |
| DWQ5 | 7198550 | 7040766 | 97.81% | 1054587575(1.05G) | 1027951836(1.03G) | 97.47% | 43.91% | 96.66% | 91.09% |
| DWQ6 | 6473366 | 6283786 | 97.07% | 948348119(0.95G) | 917432756(0.92G) | 96.74% | 43.91% | 96.76% | 91.29% |
| DWQ7 | 5927624 | 5769758 | 97.34% | 868396916(0.87G) | 842384668(0.84G) | 97.00% | 43.76% | 96.61% | 90.99% |
| DWQ8 | 6324598 | 6165240 | 97.48% | 926553607(0.93G) | 900125040(0.90G) | 97.15% | 43.81% | 96.74% | 91.26% |
| DWQ9 | 5998632 | 5872926 | 97.90% | 878799588(0.88G) | 857447196(0.86G) | 97.57% | 43.63% | 96.57% | 90.92% |
| KZE1 | 5386142 | 5237460 | 97.24% | 789069803(0.79G) | 764669160(0.76G) | 96.91% | 43.57% | 96.23% | 90.25% |
| KZE10 | 4906066 | 4598244 | 93.73% | 718738669(0.72G) | 671343624(0.67G) | 93.41% | 44.35% | 96.85% | 91.51% |
| KZE11 | 5244656 | 4981884 | 94.99% | 768342104(0.77G) | 727355064(0.73G) | 94.67% | 44.27% | 96.71% | 91.22% |
| KZE12 | 4935154 | 4604360 | 93.30% | 723000061(0.72G) | 672236560(0.67G) | 92.98% | 44.48% | 96.87% | 91.55% |
| KZE13 | 4968010 | 4703852 | 94.68% | 727813465(0.73G) | 686762392(0.69G) | 94.36% | 44.45% | 96.73% | 91.27% |
| KZE14 | 4608314 | 4369066 | 94.81% | 675118001(0.68G) | 637883636(0.64G) | 94.48% | 44.18% | 96.80% | 91.40% |
| KZE15 | 4489840 | 4208296 | 93.73% | 657761560(0.66G) | 614411216(0.61G) | 93.41% | 44.30% | 96.70% | 91.16% |
| KZE2 | 5442364 | 5305794 | 97.49% | 797306326(0.80G) | 774645924(0.77G) | 97.16% | 43.10% | 95.96% | 89.71% |
| KZE3 | 5255904 | 5075248 | 96.56% | 769989936(0.77G) | 740986208(0.74G) | 96.23% | 43.79% | 96.48% | 90.73% |
| KZE4 | 5294514 | 5154170 | 97.35% | 775646301(0.78G) | 752508820(0.75G) | 97.02% | 43.43% | 95.99% | 89.74% |
| KZE5 | 5083954 | 4960020 | 97.56% | 744799261(0.74G) | 724162920(0.72G) | 97.23% | 43.31% | 96.13% | 90.06% |
| KZE6 | 4539412 | 4272896 | 94.13% | 665023858(0.67G) | 623842816(0.62G) | 93.81% | 43.89% | 95.68% | 89.24% |
| KZE7 | 5109276 | 4957802 | 97.04% | 748508934(0.75G) | 723839092(0.72G) | 96.70% | 43.88% | 96.48% | 90.71% |
| KZE8 | 4527742 | 4293856 | 94.83% | 663314203(0.66G) | 626902976(0.63G) | 94.51% | 44.00% | 96.83% | 91.46% |
| KZE9 | 4401178 | 4122534 | 93.67% | 644772577(0.64G) | 601889964(0.60G) | 93.35% | 44.56% | 96.93% | 91.66% |
| LF1 | 4245840 | 4135560 | 97.40% | 626261400(0.63G) | 603791760(0.60G) | 96.41% | 44.10% | 96.87% | 91.31% |
| LF10 | 5259634 | 5149888 | 97.91% | 773166198(0.77G) | 751883648(0.75G) | 97.25% | 43.59% | 96.83% | 91.19% |
| LF11 | 4882236 | 4795118 | 98.22% | 717688692(0.72G) | 700087228(0.70G) | 97.55% | 43.73% | 96.88% | 91.30% |
| LF12 | 4680920 | 4594570 | 98.16% | 688095240(0.69G) | 670807220(0.67G) | 97.49% | 43.69% | 96.82% | 91.19% |
| LF13 | 5555402 | 5423478 | 97.63% | 816644094(0.82G) | 791827788(0.79G) | 96.96% | 43.88% | 96.93% | 91.42% |
| LF14 | 4840398 | 4733328 | 97.79% | 711538506(0.71G) | 691065888(0.69G) | 97.12% | 43.97% | 96.89% | 91.33% |
| LF15 | 5042168 | 4960480 | 98.38% | 741198696(0.74G) | 724230080(0.72G) | 97.71% | 43.45% | 96.95% | 91.38% |
| LF2 | 4970576 | 4861638 | 97.81% | 733159960(0.73G) | 709799148(0.71G) | 96.81% | 44.10% | 96.64% | 90.86% |
| LF3 | 5336870 | 5208508 | 97.59% | 787188325(0.79G) | 760442168(0.76G) | 96.60% | 44.02% | 96.47% | 90.44% |
| LF4 | 5638194 | 5537150 | 98.21% | 831633615(0.83G) | 808423900(0.81G) | 97.21% | 44.14% | 96.95% | 91.45% |
| LF5 | 4668206 | 4579746 | 98.11% | 688560385(0.69G) | 668642916(0.67G) | 97.11% | 44.38% | 97.14% | 91.81% |
| LF6 | 6274710 | 6183674 | 98.55% | 925519725(0.93G) | 902816404(0.90G) | 97.55% | 44.11% | 97.08% | 91.69% |
| LF7 | 4355692 | 4244376 | 97.44% | 642464570(0.64G) | 619678896(0.62G) | 96.45% | 44.29% | 97.19% | 91.96% |
| LF8 | 4658272 | 4526456 | 97.17% | 684765984(0.68G) | 660862576(0.66G) | 96.51% | 44.17% | 97.14% | 91.85% |
| LF9 | 4707358 | 4596580 | 97.65% | 691981626(0.69G) | 671100680(0.67G) | 96.98% | 44.30% | 97.11% | 91.78% |
| SF1 | 5071170 | 4903192 | 96.69% | 745461990(0.75G) | 715866032(0.72G) | 96.03% | 44.49% | 97.02% | 91.65% |
| SF10 | 4286370 | 4132984 | 96.42% | 630096390(0.63G) | 603415664(0.60G) | 95.77% | 43.79% | 96.82% | 91.21% |
| SF11 | 4146282 | 3938870 | 95.00% | 609503454(0.61G) | 575075020(0.58G) | 94.35% | 44.08% | 96.79% | 91.10% |
| SF12 | 3870212 | 3632412 | 93.86% | 568921164(0.57G) | 530332152(0.53G) | 93.22% | 44.26% | 96.82% | 91.30% |
| SF13 | 3558322 | 3325328 | 93.45% | 523073334(0.52G) | 485497888(0.49G) | 92.82% | 44.21% | 96.77% | 91.18% |
| SF14 | 3629326 | 3452442 | 95.13% | 533510922(0.53G) | 504056532(0.50G) | 94.48% | 44.02% | 96.64% | 90.91% |
| SF15 | 3643026 | 3439184 | 94.40% | 535524822(0.54G) | 502120864(0.50G) | 93.76% | 44.07% | 96.73% | 91.12% |
| SF2 | 4658088 | 4495988 | 96.52% | 684738936(0.68G) | 656414248(0.66G) | 95.86% | 44.62% | 97.04% | 91.69% |
| SF3 | 4926326 | 4823034 | 97.90% | 724169922(0.72G) | 704162964(0.70G) | 97.24% | 44.39% | 97.06% | 91.70% |
| SF4 | 4337038 | 4178448 | 96.34% | 637544586(0.64G) | 610053408(0.61G) | 95.69% | 43.72% | 96.77% | 91.09% |
| SF5 | 4464302 | 4335522 | 97.12% | 656252394(0.66G) | 632986212(0.63G) | 96.45% | 43.75% | 96.69% | 90.91% |
| SF6 | 4829066 | 4713028 | 97.60% | 709872702(0.71G) | 688102088(0.69G) | 96.93% | 43.50% | 96.47% | 90.45% |
| SF7 | 4952898 | 4830952 | 97.54% | 728076006(0.73G) | 705318992(0.71G) | 96.87% | 43.68% | 96.82% | 91.18% |
| SF8 | 4984704 | 4825852 | 96.81% | 732751488(0.73G) | 704574392(0.70G) | 96.15% | 43.69% | 96.75% | 91.06% |
| SF9 | 4658134 | 4489374 | 96.38% | 684745698(0.68G) | 655448604(0.66G) | 95.72% | 43.86% | 96.83% | 91.23% |
| TKX1 | 5844082 | 5721742 | 97.91% | 856158013(0.86G) | 835374332(0.84G) | 97.57% | 44.16% | 96.79% | 91.34% |
| TKX10 | 5178484 | 4989814 | 96.36% | 758647906(0.76G) | 728512844(0.73G) | 96.03% | 45.13% | 96.98% | 91.72% |
| TKX11 | 4206308 | 4039608 | 96.04% | 616224122(0.62G) | 589782768(0.59G) | 95.71% | 44.88% | 96.98% | 91.72% |
| TKX12 | 5319686 | 5108810 | 96.04% | 779333999(0.78G) | 745886260(0.75G) | 95.71% | 44.85% | 96.97% | 91.73% |
| TKX13 | 3941620 | 3764502 | 95.51% | 577447330(0.58G) | 549617292(0.55G) | 95.18% | 44.68% | 97.06% | 91.88% |
| TKX14 | 4997826 | 4747876 | 95.00% | 732181509(0.73G) | 693189896(0.69G) | 94.67% | 44.98% | 97.01% | 91.81% |
| TKX15 | 5227870 | 5037244 | 96.35% | 765882955(0.77G) | 735437624(0.74G) | 96.02% | 44.33% | 96.52% | 90.84% |
| TKX2 | 6194854 | 6037290 | 97.46% | 907546111(0.91G) | 881444340(0.88G) | 97.12% | 44.14% | 96.82% | 91.41% |
| TKX3 | 6850712 | 6703334 | 97.85% | 1003629308(1.00G) | 978686764(0.98G) | 97.51% | 43.98% | 96.77% | 91.30% |
| TKX4 | 6617076 | 6453284 | 97.52% | 969401634(0.97G) | 942179464(0.94G) | 97.19% | 43.98% | 96.82% | 91.43% |
| TKX5 | 5932690 | 5807894 | 97.90% | 869139085(0.87G) | 847952524(0.85G) | 97.56% | 43.99% | 96.72% | 91.20% |
| TKX6 | 5445018 | 5300656 | 97.35% | 797695137(0.80G) | 773895776(0.77G) | 97.02% | 44.21% | 96.91% | 91.59% |
| TKX7 | 5688314 | 5452050 | 95.85% | 833338001(0.83G) | 795999300(0.80G) | 95.52% | 44.74% | 96.98% | 91.76% |
| TKX8 | 5371418 | 5110688 | 95.15% | 786912737(0.79G) | 746160448(0.75G) | 94.82% | 44.80% | 96.84% | 91.51% |
| TKX9 | 5495196 | 5279328 | 96.07% | 805046214(0.81G) | 770781888(0.77G) | 95.74% | 44.73% | 97.05% | 91.91% |
| WLWT1 | 4603428 | 4465074 | 96.99% | 679005630(0.68G) | 651900804(0.65G) | 96.01% | 44.53% | 96.91% | 91.41% |
| WLWT10 | 4302242 | 4184410 | 97.26% | 634580695(0.63G) | 610923860(0.61G) | 96.27% | 44.43% | 96.92% | 91.42% |
| WLWT11 | 4848866 | 4737006 | 97.69% | 715207735(0.72G) | 691602876(0.69G) | 96.70% | 43.93% | 96.80% | 91.18% |
| WLWT12 | 4622500 | 4549732 | 98.43% | 681818750(0.68G) | 664260872(0.66G) | 97.42% | 44.36% | 96.59% | 90.60% |
| WLWT13 | 5372194 | 5210126 | 96.98% | 792398615(0.79G) | 760678396(0.76G) | 96.00% | 44.37% | 96.88% | 91.35% |
| WLWT14 | 4908044 | 4794364 | 97.68% | 723936490(0.72G) | 699977144(0.70G) | 96.69% | 44.01% | 96.70% | 90.97% |
| WLWT15 | 4023862 | 3896596 | 96.84% | 593519645(0.59G) | 568903016(0.57G) | 95.85% | 44.20% | 96.77% | 91.11% |
| WLWT2 | 4372758 | 4252598 | 97.25% | 644981805(0.64G) | 620879308(0.62G) | 96.26% | 44.67% | 96.94% | 91.45% |
| WLWT3 | 4498178 | 4420488 | 98.27% | 663481255(0.66G) | 645391248(0.65G) | 97.27% | 43.94% | 96.83% | 91.19% |
| WLWT4 | 4213696 | 4144026 | 98.35% | 621520160(0.62G) | 605027796(0.61G) | 97.35% | 43.74% | 96.40% | 90.30% |
| WLWT5 | 5037586 | 4907036 | 97.41% | 743043935(0.74G) | 716427256(0.72G) | 96.42% | 44.56% | 96.88% | 91.34% |
| WLWT6 | 4678914 | 4590134 | 98.10% | 690139815(0.69G) | 670159564(0.67G) | 97.10% | 44.11% | 96.60% | 90.73% |
| WLWT7 | 3872836 | 3740138 | 96.57% | 571243310(0.57G) | 546060148(0.55G) | 95.59% | 44.26% | 96.81% | 91.16% |
| WLWT8 | 3728822 | 3685052 | 98.83% | 550001245(0.55G) | 538017592(0.54G) | 97.82% | 43.56% | 96.55% | 90.59% |
| WLWT9 | 4362910 | 4199168 | 96.25% | 643529225(0.64G) | 613078528(0.61G) | 95.27% | 44.69% | 97.40% | 92.43% |
| WS1 | 3913820 | 3756260 | 95.97% | 575331540(0.58G) | 548413960(0.55G) | 95.32% | 43.81% | 96.39% | 90.44% |
| WS10 | 5126928 | 4931712 | 96.19% | 748531488(0.75G) | 720029952(0.72G) | 96.19% | 43.89% | 96.61% | 90.98% |
| WS11 | 4377768 | 4175168 | 95.37% | 643531896(0.64G) | 609574528(0.61G) | 94.72% | 43.88% | 96.44% | 90.71% |
| WS12 | 5344452 | 5182580 | 96.97% | 780289992(0.78G) | 756656680(0.76G) | 96.97% | 43.85% | 96.46% | 90.71% |
| WS13 | 4908312 | 4685846 | 95.47% | 721521864(0.72G) | 684133516(0.68G) | 94.82% | 44.39% | 96.97% | 91.72% |
| WS14 | 4744402 | 4551226 | 95.93% | 697427094(0.70G) | 664478996(0.66G) | 95.28% | 44.10% | 96.72% | 91.25% |
| WS15 | 5837218 | 5675554 | 97.23% | 858071046(0.86G) | 828630884(0.83G) | 96.57% | 44.28% | 96.80% | 91.39% |
| WS2 | 3358098 | 3240046 | 96.48% | 493640406(0.49G) | 473046716(0.47G) | 95.83% | 43.71% | 96.50% | 90.64% |
| WS3 | 3673094 | 3555832 | 96.81% | 539944818(0.54G) | 519151472(0.52G) | 96.15% | 43.46% | 96.49% | 90.60% |
| WS4 | 3939344 | 3765982 | 95.60% | 579083568(0.58G) | 549833372(0.55G) | 94.95% | 44.08% | 96.78% | 91.19% |
| WS5 | 5585876 | 5418280 | 97.00% | 821123772(0.82G) | 791068880(0.79G) | 96.34% | 44.24% | 96.93% | 91.66% |
| WS6 | 5036428 | 4880854 | 96.91% | 740354916(0.74G) | 712604684(0.71G) | 96.25% | 44.10% | 96.88% | 91.55% |
| WS7 | 5586868 | 5394980 | 96.57% | 821269596(0.82G) | 787667080(0.79G) | 95.91% | 44.15% | 96.61% | 91.01% |
| WS8 | 5474892 | 5296790 | 96.75% | 804809124(0.80G) | 773331340(0.77G) | 96.09% | 44.36% | 96.87% | 91.54% |
| WS9 | 4817876 | 4692902 | 97.41% | 708227772(0.71G) | 685163692(0.69G) | 96.74% | 43.59% | 96.36% | 90.51% |
