# Supplementary material for: Population Structure, Genetic Diversity and Differentiation of Triplophysa tenuis in Xinjiang Tarim River
Source: Front Genet. 2022 Mar 3;13:860678. doi: 10.3389/fgene.2022.860678 (PMC8927061; doi:10.3389/fgene.2022.860678)
Supplement: Supplementary file 1 [file DataSheet1.ZIP › Supplementary materials/Table S2.docx]

**Table S2. Genetic diversity analysis of eight *T. tenuis* populations**

| Population | Hardy-Weinberg (*HW-P*) | Expected heterozygosity (*He*) | Observed heterozygosity (*Ho*) | Polymorphism information content (*PIC*) | Observed number of alleles (*Na*) | Effective number of alleles (*Ne*) | Nucleotide diversity (*Pi*) |
| --- | --- | --- | --- | --- | --- | --- | --- |
| WLWT | 0.8777 | 0.1298 | 0.1198 | 0.1085 | 1.5584 | 1.2011 | 0.1348 |
| LF | 0.8730 | 0.1316 | 0.1210 | 0.1100 | 1.5696 | 1.2040 | 0.1365 |
| AETS | 0.8683 | 0.1358 | 0.1249 | 0.1136 | 1.5854 | 1.2105 | 0.1410 |
| SF | 0.8551 | 0.1449 | 0.1307 | 0.1207 | 1.6032 | 1.2268 | 0.1505 |
| WS | 0.8495 | 0.1450 | 0.1295 | 0.1214 | 1.6172 | 1.2241 | 0.1508 |
| DWQ | 0.8734 | 0.1325 | 0.1235 | 0.1113 | 1.5951 | 1.2030 | 0.1373 |
| TKX | 0.8695 | 0.1343 | 0.1238 | 0.1130 | 1.6099 | 1.2055 | 0.1393 |
| KZE | 0.8705 | 0.1336 | 0.1228 | 0.1124 | 1.6057 | 1.2046 | 0.1386 |
